# Practical Notes on Diagnosis and Treatment

**Published:** 1907-06-22

**Authors:** 


### Practical Notes on Diagnosis and Treatment.

**Delirium Tremens.**

Dr. Bellamy reports that the delirium in a series of cases of delirium tremens was more rapidly and promptly controlled by trional than by any other hypnotic.

**Warts.**

The following is a formula recommended by Kaposi: Sublimed sulphur 20 parts, glycerine 50 parts, acetic acid 10 parts. Mix, and paint on the warts several times daily.

**Use of Ice in Asthma.**

A severe attack of bronchial asthma may sometimes be cut short by the application of a piece of ice over the course of the pneumogastric nerve at the root of the neck.

**Paraplegia.**

The fact that both limbs are equally paralysed suggests a lesion in the spinal cord. With a brain lesion there would be unilateral paralysis, whilst in a peripheral neuritis it is common to find that the two limbs are not equally affected.

**Prognosis in Spinal Abscess.**

In cases of psoas abscess—indeed, in all cases of spinal abscess, whether psoas or lumbar—the prognosis becomes more and more unfavourable as the patients pass to the wrong side of 25 years of age. *Sir Wm. Bennett.*

**Molluscum Contagiosum.**

To cure this, touch the top of each lesion with a drop of pure carbolic acid; after a few applications the small waxy-like tumour withers and falls off. The diagnosis is easy from the presence of a central depression in the little button-like growth.

**Disseminated Sclerosis.**

It is a very good thing when you recognise a case of disseminated sclerosis to put the patient in bed and keep him there for two or three months, absolutely at rest. Another thing that often does good is the combination of rest with massage, and you may use Faradism also.—*Dr. James Taylor.*

**Bow Legs.**

Bow-legs are developed only in the rickety subject. The deformity depends upon a yielding of the softened diaphyses of the femur and tibia separately, and to some extent independently of one another, and in a vertical transverse plane during the period that the child is learning to stand.—*Mr. Arbuthnot Lane.*

**The Age Incidence of Aneurysm.**

There is a sort of idea abroad that aneurysm is a disease of old people; but practically that is not so. Aneurysm is really a disease of the prime of life. Therefore you must never be surprised to find an aneurysm of the aorta in persons so young, say, as five-and-twenty years of age.—*Dr. Goodhart.*

**Hernia in Young Women.**

It is not uncommonly supposed to be almost proof positive that the ovary is in the sac of a hernia, if the pain and tenderness about the hernia are increased at the menstrual epoch. The idea is a pure fallacy. Indeed, such conditions may exist even when there is nothing at all in the sac except fluid.—*Sir Wm. Bennett.*

**Grey Powder.**

I want you to avoid the popular error which causes infants and young children to be treated with "grey powder" whenever their tongues are coated or the colour of their motions is green, the idea being that their livers are, by these symptoms, shown to be out of order and to need some touching up.—*Dr. Allan Jamieson.*

**Epilepsy and Intra-Cranial Tumour.**

Among the initial symptoms of cerebral tumour is the occurrence of an attack of epilepsy. The recurrence of a series of such attacks may cause the physician to believe that he is dealing with a case of idiopathic epilepsy. Hence when these circumstances exist the possibility of tumour should be borne in mind.

**Syphilitic Deafness.**

Attacks of syphilitic deafness, depending probably on inflammation of the fibrous structures in the internal and middle ear, are not very uncommon, and a very severe pain in the head is a frequent concomitant. Facial paralysis is rare in such cases, but a few examples of it have been recorded.—*Mr. Jonathan Hutchinson.*

**Treatment in Cirrhosis of the Liver.**

Paracentesis should be performed as soon as the ascites is sufficient to exercise injurious pressure, and perhaps marked diminution in the quantity of the urine is the most reliable indication of this. So long as the ascitic fluid obstructs the renal circulation by pressure on the renal veins diuretics will be inert; it is after paracentesis that these remedies becomes useful.—*Dr. T. H. Green.*

